# Inflammation Shapes Stem Cells and Stemness during Infection and Beyond

**DOI:** 10.3389/fcell.2016.00118

**Published:** 2016-11-02

**Authors:** Stella Michael, Charis Achilleos, Theofano Panayiotou, Katerina Strati

**Affiliations:** Department of Biological Sciences, University of CyprusNicosia, Cyprus

**Keywords:** inflammation, stem cells, stemness, stem cells and sterile inflammation, stem cells and microbes

## Abstract

The outcome of an inflammatory incident can hang in the balance between restoring health and tissue integrity on the one hand, and promoting aberrant tissue homeostasis and adverse outcomes on the other. Both microbial-related and sterile inflammation is a complex response characterized by a range of innate immune cell types, which produce and respond to cytokine mediators and other inflammatory signals. In turn, cells native to the tissue in question can sense these mediators and respond by migrating, proliferating and regenerating the tissue. In this review we will discuss how the specific outcomes of inflammatory incidents are affected by the direct regulation of stem cells and cellular plasticity. While less well appreciated than the effects of inflammatory signals on immune cells and other differentiated cells, the effects are crucial in understanding inflammation and appropriately managing therapeutic interventions.

## Introduction

Inflammation has a well-established role in the defense of organisms against microbial invasion. The presence of commensal and pathogenic microbes, both intracellular and extracellular, is usually detected by receptors residing mostly in the surface of innate immune cells known as pattern recognition receptors (PRRs). These receptors are the first line of the surveillance system which ultimately recognizes pathogen associated molecular patterns (PAMPs) and triggers the inflammatory response. Acute and chronic inflammation in a number of diseases associated with pathogens and the interplay between infection and inflammation is of paramount importance to clinical outcomes (Apidianakis and Ferrandon, 2014).

While inflammation is a defense against pathogens it can also be triggered during processes unrelated to microbial insult. This process, termed sterile inflammation, is typically linked to chemical or physical triggers. Inflammatory cells present at the site of the damage recognize danger-associated molecular patterns (DAMPs) and secrete molecules which prime the tissue restoration via the proliferation of quiescent adult stem cells (Nagaoka et al., 2000; Koh and DiPietro, 2011; Petrie et al., 2014; Kizil et al., 2015). Sterile inflammation can have profound effects on tissue homeostasis and repair, for example during wound healing, or during the onset and initiation of inflammatory diseases.

**Figure 1:**
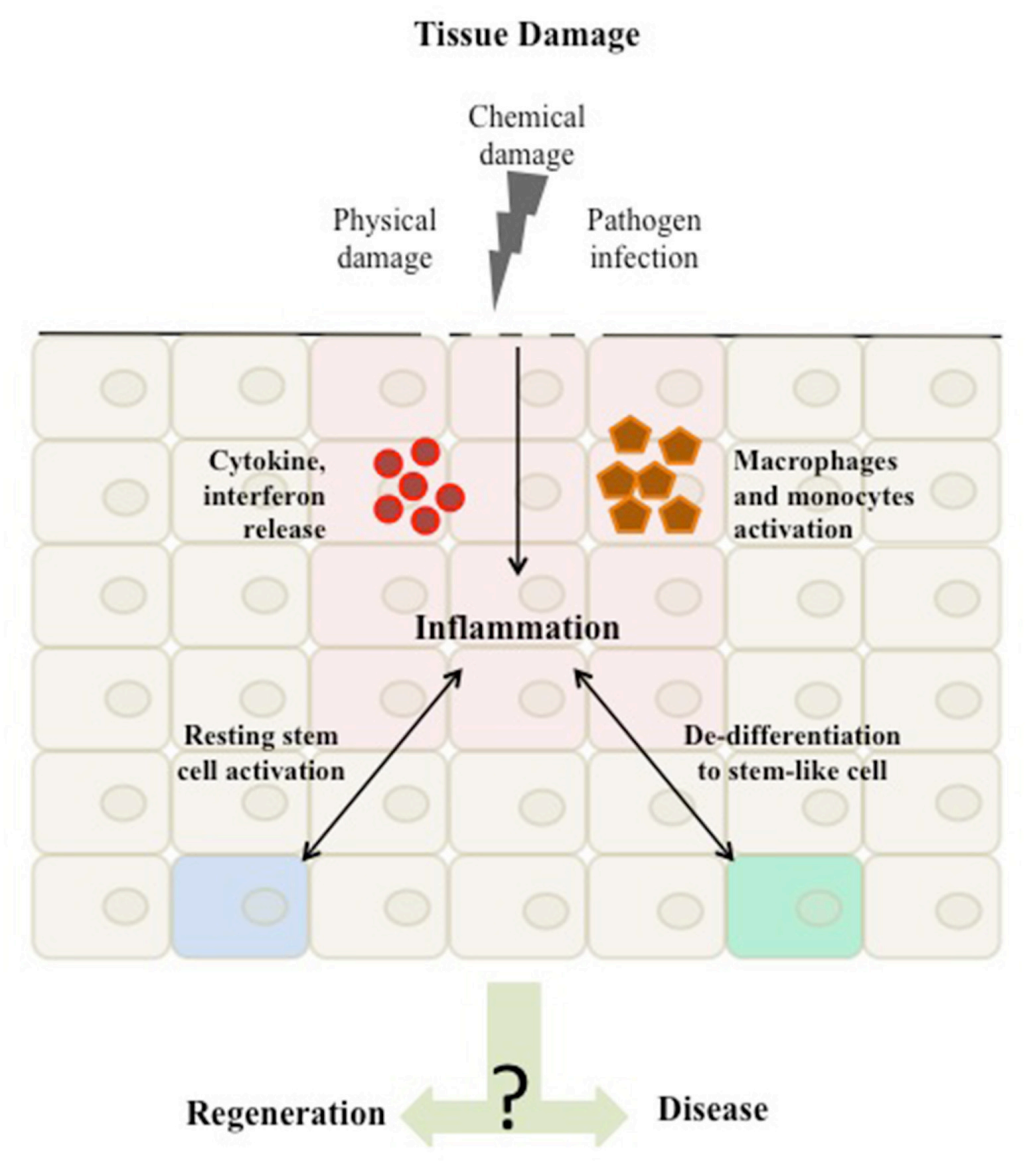
**Tissue damage can arise as a result of physical damage, chemical damage or pathogen infection**. Once the damage is detected a homeostatic inflammatory response is activated to regenerate the tissue. This inflammation is characterized by the activation of immune cells, such as macrophages and monocytes and the release of inflammatory mediators, such as cytokines and interferons to the site of damage. These mediators in turn can affect native tissue cells to respond by migrating and proliferating resulting in tissue repair. Evidence suggest that the inflammatory response acts as a regulator of tissue stemness either by directly affecting tissue stem cells or by shifting differentiated cells toward a stem-like cell character. The balance in this inflammatory response and its mediated stemness is a critical driver of either maintaining tissue integrity or promoting aberrant homeostasis and disease.

We summarize here evidence for the direct crosstalk between the inflammatory response and stem cells both in cases of microbial and sterile induced inflammation (Figure 1). Inflammation is emerging as an important regulator of stem cells and plays an intricate role in health and disease.

## Regulation of stem cells in response to microbial motifs

Recently PRRs were shown to be expressed in the surface of tissue stem cells suggesting that there is the potential for direct effects of PAMPs on stem cell behavior (Boiko and Borghesi, 2012). For example, hematopoietic stem cells express toll-like receptors (TLRs) whose activation leads to the differentiation of myeloid progenitors into monocytes and macrophages immune cells (Nagai et al., 2006) as seen in the presence of the vaccinia virus in the bone marrow (Singh et al., 2008). Stem cells of solid tissues, more prominently the gut, have also been shown to express PRRs integrating inflammation to immune clearance and subsequent tissue regeneration. Intestinal stem cells (ISCs) expressing TLR4 show increased proliferation and expansion of the stem cell population in the intestinal epithelium (Santaolalla et al., 2013). ISCs have also been shown to express Nod2, a general sensor for peptidoglycan (Girardin et al., 2003). The constitutive expression of Nod2, in ISCs, provides protection against stress (Nigro et al., 2014). The ability of gut stem cells to respond directly to patterns, such as LPS (via TLR4) and peptidoglycan (via Nod2), may underlie mechanisms of tissue response not only to pathogenic bacteria but also commensals and is likely important to general tissue homeostasis via the interaction with intestinal microbiota. The findings in mammalian systems are corroborated by extensive literature in other experimental systems, such as Drosophila (Panayidou and Apidianakis, 2013).

Once an inflammatory program has already been initiated the production of cytokines, interferons etc. by local immune populations can further impact the behavior of stem cells. In the gut, innate lymphoid cells produce interleukin-22, a potent survival factor, which can directly act on ISCs promoting growth and epithelial regeneration (Lindemans et al., 2015). Chronic HBV infection also stimulates release of interleukin-22 by inflammatory cells, inducing proliferation of liver stem cells (Feng et al., 2012). In addition, the mediator of inflammation TNF-α is activated as a result of brain inflammation in neural stem cells seen in conditions of trauma, multiple sclerosis and pathogen infections. TNF-α activation ultimately brings about proliferation of the neural stem cells (Widera et al., 2006). Neural stem cells in the hippocampus have also been shown by *in vivo* studies to proliferate upon the presence of bacterial enterotoxins (Wolf et al., 2009). In the urinary tract, upon *E. coli* infection, the uroepithelial stem cells are activated for epithelial renewal in response to the inflammatory response (Mysorekar et al., 2009).

The presence of pathogenic burden in the hematopoietic system rapidly depletes immune cells stimulating intermediate blood progenitors to maintain blood cell balance (Hawkins et al., 2006). Inflammation-induced myelopoiesis, due to pathogen presence, results in the release of interleukin-27 causing activation and differentiation of hematopoietic stem cells (HSCs) (Furusawa et al., 2016). Chronic infection, as seen in the presence of the *Mycobacterium avium*, results in the activation of quiescent HSCs through the release of the inflammatory mediator interferon-γ (Baldridge et al., 2010).

Inflammation is proposed to promote tissue recovery via its effects on differentiated cells to regenerate the tissue (Karin and Clevers, 2016). In some cases the differentiated cells de-differentiate in response to inflammation, acquiring stem-like characteristics and increased cellular plasticity. In support of this idea, the induction of immunity was found to be required for efficient nuclear reprogramming *in vitro* (Lee et al., 2012).

Tissue reprogramming is achieved through the upregulation of growth factors and cytokines in the inflammatory microenvironment (Grivennikov et al., 2010). This could be attributed to changes in the expression of specific genes/pathways which shift a differentiated cell closer toward a stem cell character. Alternatively, the effects could impart tissue stem cells or progenitors with increased/altered capabilities.

There are a number of examples to support the idea that inflammation caused by infections leads to tissue regeneration and/or cellular stemness. Such a response has been observed in viral infections of HBV and HCV where inflammation in the liver induced expression of stemness markers (Karakasiliotis and Mavromara, 2015). Specifically, the secretion of interleukin 6 (IL6) by the inflammatory cells during HBV infection regulates the expression of Oct4 and Nanog pluripotency factors (Chang et al., 2015). Furthermore, the hypoxia factor HIF-1α produced in the HCV virally-infected cells confers an epithelial-mesenchymal transition (EMT) character (Wilson et al., 2012). It is important to note that in this case, the EMT is accompanied by enhanced viral replication.

In fact, inflammation-mediated changes on the differentiation status of the tissue are a factor in the pathology which accompanies disease. Persistent induction of stemness in the infected tissue in the presence of chronic inflammation, as seen in infections of the gut, can likely contribute to carcinogenesis (Apidianakis and Ferrandon, 2014; Kuo et al., 2016). However, emerging concept suggests that it may also be beneficial to the pathogen. Several groups have shown that for some infectious agents it can play a role during their replication (as in the case of HBV and HCV), their dissemination, and protection (Masaki et al., 2013; Nigro et al., 2014; Karakasiliotis and Mavromara, 2015). A more prominent example, the leprosy bacterium infects preferentially Schwann cells of the nervous system and induces their reprogramming into stem-like cells. The infected stem-like cells then migrate to the mesenchyme where they re-differentiate to mesenchyme tissue allowing for expansion of the infection (Masaki et al., 2013). The innate immune response has been shown to precede this reprogramming (Masaki et al., 2014). For efficient dissemination, the cells need to evade the host immunity and they do so by inducing an inflammatory response achieved through the release of factors from the stem-like cells. This subsequently recruits macrophages that form granulomas able to bypass immunity and migrate.

The inflammatory response is important to the host organism as a protective mechanism against pathogen invasion as well as tissue regeneration through the induction of stemness. In some cases however, through inflammation, pathogens are able to escape immune surveillance for their protection and dissemination with possible consequences to their lifecycle and replicative potential, as we have seen in the cases of the HBV and HCV viruses or of bacteria in the intestine or the nervous system.

## Regulation of stem cells during tissue (re)generation

Sterile inflammation has also been shown to lead to profound changes in the differentiation status of the tissue. An example is the regenerative process which takes place in response to wound healing. Wound healing requires an ordered sequence of events ranging from acute inflammation, tissue organization, and remodeling (Gurtner et al., 2008; Karin and Clevers, 2016). However, the period of tissue repair varies between the extent of the damage and the site of the damaged tissue (Meyer et al., 1992; Gordon et al., 2003; Pitsouli et al., 2009).

Tissue repair following an insult restores health in the tissue and preserves the state of homeostasis. Regeneration in the tissue is achieved via the priming of resident slow-cycling stem cells to adopt a proliferative state and yield transit-amplifying cells which will differentiate to restore tissue architecture. Sterile inflammation plays an important role in this process in ways which are likely distinct to those seen during infection (Bezbradica et al., 2016).

The intestine can regenerate very rapidly. Tissue restoration is mediated via neutrophil infiltration at the site of the damage. These are responsible for JNK activation and the priming and proliferation of slow–cycling ISCs (Karin and Clevers, 2016). Work from Riehl et al. (2000) further supported the role of inflammatory signals, as administration of inflammatory cytokines and growth factors following radiation exposure rescued damaged epithelium via the proliferation and differentiation of intestinal stem cells (Riehl et al., 2000). However, homeostasis is sustained via the activation of developmental and inflammatory pathways which activate dormant ISCs. For instance, JNK signaling pathway is responsible for the secretion of IL6 which activates the JAK-STAT pathways leading to ISC proliferation (Jiang et al., 2009; Liu et al., 2010; Kuhn et al., 2014).

In the case of the skin epithelium, which also encounters frequent damage, it is proposed that keratinocytes trigger the inflammatory phase of the wound healing by secreting molecules like IL6 and TNFα (Wang et al., 2004; Ryser et al., 2014; Rittié, 2016). These inflammatory molecules along with other developmental mediators, such as extracellular matrix components (Kurbet et al., 2016) signal to the niche of slow-cycling adult stem cells in order to train them toward proliferation and ultimate differentiation (Cotsarelis et al., 1990; Taylor et al., 2000).

Another organ that has a fast-paced regeneration with the ability to recover its mass even after substantial loss is the liver (Michalopoulos, 2007). While it remains controversial whether the adult liver depends on a stem cell population during homeostatic conditions, various stem cell or progenitor populations have been described to participate in its regeneration following injury (Yimlamai et al., 2014; Wang et al., 2015). Soon after partial hepatectomy, the inflammatory cytokines TNF-α and IL6 are upregulated at the site of regeneration guiding hepatocytes to enter mitosis and restore lost tissue. Beyond the involvement of unipotent mature hepatocytes, which assist in the repair process of the liver particularly in response to acute inflammation, liver progenitor cells are also present to mediate liver repair. This is typically the case in the context of chronic inflammation, when mature hepatocytes have reached their replicative limit (Viebahn and Yeoh, 2008; Español-Suñer et al., 2012). While some controversy exists with regard to the exact characteristics of liver progenitors (Dollé et al., 2010), they respond to inflammatory signals following injury to generate differentiated cells essential for liver regeneration.

Our understanding of the effects of inflammatory signaling on stem cells, stems mostly from model tissue systems, such as the intestine, skin and liver. However, the inflammatory signaling to resident tissue stem cells is corroborated as crucial to regeneration in more poorly understood tissues and non-mammalian model systems as well (Apidianakis and Rahme, 2011). In mice, the recently described dclk1 progenitors have been shown to respond to inflammation during pancreatic regeneration (Westphalen et al., 2016). There is also extensive work implicating crosstalk between neural stem cells and inflammation in mammals and zebrafish (Kizil et al., 2015). Specifically, during zebrafish brain regeneration, inflammation is the trigger which initiates neural stem cell proliferation (Kyritsis et al., 2012).

While inflammation can prime stem cell responses it is becoming increasingly clear that in certain contexts stem cells possess immunomodulatory potential. Mesenchymal stem cells and neural stem cells are the two cell types most often ascribed with immunomodulatory potential (Kizil et al., 2015; Le Blanc and Davies, 2015). In both these examples stem cells have been shown to dampen or alter inflammation with beneficial outcomes in inflammation-associated disease. Despite the fact that the mechanisms are still poorly understood there is justified excitement for the potential application of these properties in therapeutics.

The crosstalk between sterile inflammation and stem cell plasticity within a tissue during the wound healing response is a critical step in the regenerative process. Priming stem cells that reside in the circulation, in addition to the stem cells of the regenerating tissue, may also contribute to this process. HSCs are likely directed to liver and skin following physical damage (Rennert et al., 2012). It was further suggested that inflammatory cytokines and growth factors released due to tissue injuries can stimulate a signaling wave toward bone marrow-residing stem cells to enter the circulation and inhabit the injured site. These bone-marrow stem cells and locally residing tissue stem cells hold the capacity of tissue regeneration. Perhaps more surprisingly, sterile inflammatory signaling, such as that initiated by IFNγ and TLR4, plays a role not only in the regeneration of adult tissue but is a well-conserved regulator in their production during development (Li et al., 2014; He et al., 2015). These findings certainly reframe currently held ideas about the evolutionary function of inflammation.

## Inflammatory signaling during disease

In some cases inflammation, particularly where it is chronic, can lead to the development of disease. The continuous and often aberrant response of stem cells as a result of this signaling has been shown to play an important part in this process. Intestinal stem cells express TLR4 the activation of which can lead to ER-stress, a trigger for stem cell apoptosis during necrotizing enterocolitis (Afrazi et al., 2014). In Barrett's esophagus Lgr5^+^ gastric cardia stem cells can migrate in response to the inflammatory signaling and are the likely source of the metaplastic and dysplastic cells observed in the course of the disease (Quante et al., 2012).

The disease most commonly associated with inflammation is of course cancer. Multiple studies have produced substantial evidence suggesting that cancer and inflammation are in many cases connected, interdependent biological processes (Coussens and Werb, 2002; Balkwill et al., 2005; Karin, 2006). This is true in cases where the cancer is associated with microbial or viral causes, but also in cases where no pathogen is directly linked. For example, in addition to their mutagenic effects, carcinogens in tobacco smoke cause damage and chronic inflammation to the lungs and increase the risk of cancer development (Punturieri et al., 2009). Autoimmunity is also associated with increased tumor development. The chance of developing colitis-associated cancer or lymphoma is increased in people suffering from inflammatory bowel or celiac disease, respectively (Kraus and Arber, 2009; Waldner and Neurath, 2009).

The important bidirectional link between inflammation and stem cells has direct implications on cancer development. Studies have suggested that HSC recruitment and differentiation is directly linked to increased inflammation. For example, CD34+ progenitor cells are recruited to sites with increased inflammation, probably using the same adhesion and chemokine receptors used for stem cell homing to the bone marrow (PSGL-1, CXCL12, α4β1 integrin, CD44, and others) (Blanchet and McNagny, 2009). Inflammatory mediators seem to have a vital role in inducing expression of stemness-related genes. The expression of stemness-related genes in cancer is likely linked to the generation and evolution of the compartment of cells able to regenerate tumor diversity, the cancer stem cells (CSCs) (Kuo et al., 2016; Uthaya Kumar et al., 2016). Upregulation of OCT4 has been shown to contribute to tumor cell migration and resistance to cancer therapeutics (Ma et al., 2011; Chang et al., 2015, 2016; Bhatt et al., 2016).

While inflammation is one of the stimuli suggested to initiate such transcriptional changes, once CSCs form, evidence suggests that they can serve to further amplify inflammatory signaling. Chemoresistant CSCs were found to express proinflammatory gene signatures, mainly due to the sustained activation of NF-kB and interferon-stimulated regulatory element (ISRE)-dependent pathways. Notably, tumor-associated macrophages in this environment protect tumor cells from chemotherapeutic agents by promoting and enhancing the tumor growth properties of CSCs (Jinushi et al., 2011). A proinflammatory signature has also been exhibited by leukemia stem cells which can promote chemoresistance by means of metabolic adaptation (Ye et al., 2016). Thus, in some cases, tumors respond to chemotherapy by altering the immunological profile of the microenvironment in part due to the direct action of CSCs, thus further enabling tumor growth (Jinushi et al., 2011; Jinushi, 2014).

In more rare cases recruited circulating stem cells, subjected to chronic inflammation in a tissue, have been proposed to act as cancer-initiating cells themselves. Houghton et al. (2004), used a mouse model to demonstrate an extremely important connection between chronic inflammation, hematopoietic stem-cell recruitment and cancer development at the site of inflammation (Houghton et al., 2004). In this study, infection by *Helicobacter pylori* in the mouse caused the recruitment and subsequent engraftment of bone marrow derived stem cells (BMDC) into the stem cell compartment of the gastric mucosa. Within their new, inflamed niche, these engrafted stem cells accumulated mutations, and eventually gave rise to gastric tumors. This study showcased a direct connection between chronic inflammation, stem cell recruitment and increased cancer development (Houghton et al., 2004).

## Conclusion

Inflammatory signaling promotes cellular responses with critical ramifications during infection, tissue generation/regeneration, cancer and other diseases. We summarize here work which demonstrates that stem cells can respond to and participate in inflammatory cascades in a direct manner. They express receptors which detect PAMPs and DAMPs, the initial triggers of the inflammatory response. They are able to mobilize and proliferate in response to inflammation in addition to producing cytokines which further amplify the response. Accumulating evidence suggests that in cases where a pathogen is involved, the changes in stemness mediated by inflammation also have a profound influence on the lifecycle of the pathogen. This is an area which merits further research. Understanding the crosstalk between stem cells and inflammation is an important piece of the puzzle which refines our understanding of the evolutionary roles of inflammation. Furthermore, it provides indispensable tools in our quest to harness knowledge into useful therapeutics.

## Author contributions

SM, CA, and TP performed literature searches, and co-wrote the review. These three authors contributed equally. KS conceived the mini review topic, performed literature searches, and co-wrote the review.

## Funding

Research in the laboratory of KS is currently funded by the University of Cyprus.

### Conflict of interest statement

The authors declare that the research was conducted in the absence of any commercial or financial relationships that could be construed as a potential conflict of interest.
